# Supplementary material for: GenePattern flow cytometry suite
Source: Source Code Biol Med. 2013 Jul 3;8:14. doi: 10.1186/1751-0473-8-14 (PMC3717030; doi:10.1186/1751-0473-8-14)
Supplement: Additional file 1 — Example of a quality assessment report. Please use any ZIP-compatible software to extract the ZIP archive file into a folder and then open the index.html file in your web browser. The interactive report shows an example of quality assessment with samples in rows and performed tests in columns. The results are color-coded with green indicating no problems, yellow indicating a warning, and red suggesting the failure of a certain test on a certain sample. The user should review flagged samples and decide whether further actions are required. Clicking on a heading shows an overview plot for a particular test. Clicking on a ‘+’ sign will expand the appropriate section, revealing detailed test results. Individual dots can be clicked on to provide the supporting analyses of tested FCM parameters underlying the final call. The example demonstrates a quality assessment report of a 96 well plate of a “Normal Donor” study performed by Becton, Dickinson and Company (BD) in order to measure immune responses to various infectious agents and cancer antigens among healthy young adults. The ≈ 8 GB of data from the mentioned study may be downloaded from http://www.ficcs.org/data/data-files/. [file 1751-0473-8-14-S1.zip › AdditionalFile1_QAReportExample/reports/index.html]

qatest


| flow set details | cell number | median | | | probabilty density function | | | emprical cumulative distribution function | | | boxplot | | | time line | | time flow |
| --- | --- | --- | --- | --- | --- | --- | --- | --- | --- | --- | --- | --- | --- | --- | --- | --- |
| name \| desc \| minRange \| maxRange; FSC-H \| FSC-Height \| 0 \| 1023; SSC-H \| SSC-Height \| 0 \| 1023; FL1-H \| IFNg FITC \| 1 \| 1023; FL2-H \| CD69 PE \| 1 \| 1023; FL3-H \| CD4 PerCP Cy5.5 \| 1 \| 1023; FL1-A \| NA \| 0 \| 1023; FL4-H \| CD3 APC \| 1 \| 1023; Time \| Time (102.40 sec.) \| 0 \| 1023 |  |  | | |  | | |  | | |  | | |  | |  |
| 1 msi031904150.A01 name; msi031904150.A01 | 43000 | +  – | FSC-H 380 | SSC-H 110 | +  – | FSC-H | SSC-H | +  – | FSC-H | SSC-H | +  – | FSC-H | SSC-H | +  – | FSC-H 0 |  |
|  |  |  |  |  |  |  |  |  |  |  |  |  |  |  |  |  |
| 2 msi031904150.A02 name; msi031904150.A02 | 47000 |  | FSC-H 380 | SSC-H 110 |  | FSC-H | SSC-H |  | FSC-H | SSC-H |  | FSC-H | SSC-H |  | FSC-H 0 |  |
|  |  |  |  |  |  |  |  |  |  |  |  |  |  |  |  |  |
| 3 msi031904150.A03 name; msi031904150.A03 | 44000 |  | FSC-H 380 | SSC-H 100 |  | FSC-H | SSC-H |  | FSC-H | SSC-H |  | FSC-H | SSC-H |  | FSC-H 0 |  |
|  |  |  |  |  |  |  |  |  |  |  |  |  |  |  |  |  |
| 4 msi031904150.A04 name; msi031904150.A04 | 45000 |  | FSC-H 380 | SSC-H 100 |  | FSC-H | SSC-H |  | FSC-H | SSC-H |  | FSC-H | SSC-H |  | FSC-H 0 |  |
|  |  |  |  |  |  |  |  |  |  |  |  |  |  |  |  |  |
| 5 msi031904150.A05 name; msi031904150.A05 | 46000 |  | FSC-H 390 | SSC-H 110 |  | FSC-H | SSC-H |  | FSC-H | SSC-H |  | FSC-H | SSC-H |  | FSC-H 0 |  |
|  |  |  |  |  |  |  |  |  |  |  |  |  |  |  |  |  |
| 6 msi031904150.A06 name; msi031904150.A06 | 45000 |  | FSC-H 390 | SSC-H 110 |  | FSC-H | SSC-H |  | FSC-H | SSC-H |  | FSC-H | SSC-H |  | FSC-H 0 |  |
|  |  |  |  |  |  |  |  |  |  |  |  |  |  |  |  |  |
| 7 msi031904150.A07 name; msi031904150.A07 | 45000 |  | FSC-H 380 | SSC-H 110 |  | FSC-H | SSC-H |  | FSC-H | SSC-H |  | FSC-H | SSC-H |  | FSC-H 0 |  |
|  |  |  |  |  |  |  |  |  |  |  |  |  |  |  |  |  |
| 8 msi031904150.A08 name; msi031904150.A08 | 44000 |  | FSC-H 390 | SSC-H 110 |  | FSC-H | SSC-H |  | FSC-H | SSC-H |  | FSC-H | SSC-H |  | FSC-H 0 |  |
|  |  |  |  |  |  |  |  |  |  |  |  |  |  |  |  |  |
| 9 msi031904150.A09 name; msi031904150.A09 | 44000 |  | FSC-H 380 | SSC-H 110 |  | FSC-H | SSC-H |  | FSC-H | SSC-H |  | FSC-H | SSC-H |  | FSC-H 0 |  |
|  |  |  |  |  |  |  |  |  |  |  |  |  |  |  |  |  |
| 10 msi031904150.A10 name; msi031904150.A10 | 43000 |  | FSC-H 390 | SSC-H 110 |  | FSC-H | SSC-H |  | FSC-H | SSC-H |  | FSC-H | SSC-H |  | FSC-H 0 |  |
|  |  |  |  |  |  |  |  |  |  |  |  |  |  |  |  |  |
| 11 msi031904150.A11 name; msi031904150.A11 | 44000 |  | FSC-H 400 | SSC-H 110 |  | FSC-H | SSC-H |  | FSC-H | SSC-H |  | FSC-H | SSC-H |  | FSC-H 0 |  |
|  |  |  |  |  |  |  |  |  |  |  |  |  |  |  |  |  |
| 12 msi031904150.A12 name; msi031904150.A12 | 44000 |  | FSC-H 400 | SSC-H 110 |  | FSC-H | SSC-H |  | FSC-H | SSC-H |  | FSC-H | SSC-H |  | FSC-H 0 |  |
|  |  |  |  |  |  |  |  |  |  |  |  |  |  |  |  |  |
| 13 msi031904150.B01 name; msi031904150.B01 | 44000 |  | FSC-H 390 | SSC-H 110 |  | FSC-H | SSC-H |  | FSC-H | SSC-H |  | FSC-H | SSC-H |  | FSC-H 0 |  |
|  |  |  |  |  |  |  |  |  |  |  |  |  |  |  |  |  |
| 14 msi031904150.B02 name; msi031904150.B02 | 45000 |  | FSC-H 380 | SSC-H 110 |  | FSC-H | SSC-H |  | FSC-H | SSC-H |  | FSC-H | SSC-H |  | FSC-H 0 |  |
|  |  |  |  |  |  |  |  |  |  |  |  |  |  |  |  |  |
| 15 msi031904150.B03 name; msi031904150.B03 | 41000 | +  – | FSC-H 380 | SSC-H 100 | +  – | FSC-H | SSC-H | +  – | FSC-H | SSC-H | +  – | FSC-H | SSC-H | +  – | FSC-H 0 |  |
|  |  |  |  |  |  |  |  |  |  |  |  |  |  |  |  |  |
| 16 msi031904150.B04 name; msi031904150.B04 | 44000 |  | FSC-H 390 | SSC-H 100 |  | FSC-H | SSC-H |  | FSC-H | SSC-H |  | FSC-H | SSC-H |  | FSC-H 0 |  |
|  |  |  |  |  |  |  |  |  |  |  |  |  |  |  |  |  |
| 17 msi031904150.B05 name; msi031904150.B05 | 43000 |  | FSC-H 390 | SSC-H 110 |  | FSC-H | SSC-H |  | FSC-H | SSC-H |  | FSC-H | SSC-H |  | FSC-H 0 |  |
|  |  |  |  |  |  |  |  |  |  |  |  |  |  |  |  |  |
| 18 msi031904150.B06 name; msi031904150.B06 | 44000 |  | FSC-H 390 | SSC-H 110 |  | FSC-H | SSC-H |  | FSC-H | SSC-H |  | FSC-H | SSC-H |  | FSC-H 0 |  |
|  |  |  |  |  |  |  |  |  |  |  |  |  |  |  |  |  |
| 19 msi031904150.B07 name; msi031904150.B07 | 45000 |  | FSC-H 390 | SSC-H 100 |  | FSC-H | SSC-H |  | FSC-H | SSC-H |  | FSC-H | SSC-H |  | FSC-H 0 |  |
|  |  |  |  |  |  |  |  |  |  |  |  |  |  |  |  |  |
| 20 msi031904150.B08 name; msi031904150.B08 | 45000 |  | FSC-H 390 | SSC-H 110 |  | FSC-H | SSC-H |  | FSC-H | SSC-H |  | FSC-H | SSC-H |  | FSC-H 0 |  |
|  |  |  |  |  |  |  |  |  |  |  |  |  |  |  |  |  |
| 21 msi031904150.B09 name; msi031904150.B09 | 44000 |  | FSC-H 390 | SSC-H 110 |  | FSC-H | SSC-H |  | FSC-H | SSC-H |  | FSC-H | SSC-H |  | FSC-H 0 |  |
|  |  |  |  |  |  |  |  |  |  |  |  |  |  |  |  |  |
| 22 msi031904150.B10 name; msi031904150.B10 | 42000 |  | FSC-H 380 | SSC-H 100 |  | FSC-H | SSC-H |  | FSC-H | SSC-H |  | FSC-H | SSC-H |  | FSC-H 0 |  |
|  |  |  |  |  |  |  |  |  |  |  |  |  |  |  |  |  |
| 23 msi031904150.B11 name; msi031904150.B11 | 46000 |  | FSC-H 390 | SSC-H 110 |  | FSC-H | SSC-H |  | FSC-H | SSC-H |  | FSC-H | SSC-H |  | FSC-H 1.1 |  |
|  |  |  |  |  |  |  |  |  |  |  |  |  |  |  |  |  |
| 24 msi031904150.B12 name; msi031904150.B12 | 42000 |  | FSC-H 390 | SSC-H 100 |  | FSC-H | SSC-H |  | FSC-H | SSC-H |  | FSC-H | SSC-H |  | FSC-H 0 |  |
|  |  |  |  |  |  |  |  |  |  |  |  |  |  |  |  |  |
| 25 msi031904150.C01 name; msi031904150.C01 | 45000 |  | FSC-H 380 | SSC-H 100 |  | FSC-H | SSC-H |  | FSC-H | SSC-H |  | FSC-H | SSC-H |  | FSC-H 0 |  |
|  |  |  |  |  |  |  |  |  |  |  |  |  |  |  |  |  |
| 26 msi031904150.C02 name; msi031904150.C02 | 45000 |  | FSC-H 390 | SSC-H 100 |  | FSC-H | SSC-H |  | FSC-H | SSC-H |  | FSC-H | SSC-H |  | FSC-H 0 |  |
|  |  |  |  |  |  |  |  |  |  |  |  |  |  |  |  |  |
| 27 msi031904150.C03 name; msi031904150.C03 | 42000 |  | FSC-H 390 | SSC-H 100 |  | FSC-H | SSC-H |  | FSC-H | SSC-H |  | FSC-H | SSC-H |  | FSC-H 0 |  |
|  |  |  |  |  |  |  |  |  |  |  |  |  |  |  |  |  |
| 28 msi031904150.C04 name; msi031904150.C04 | 46000 |  | FSC-H 390 | SSC-H 100 |  | FSC-H | SSC-H |  | FSC-H | SSC-H |  | FSC-H | SSC-H |  | FSC-H 0 |  |
|  |  |  |  |  |  |  |  |  |  |  |  |  |  |  |  |  |
| 29 msi031904150.C05 name; msi031904150.C05 | 44000 | +  – | FSC-H 390 | SSC-H 110 | +  – | FSC-H | SSC-H | +  – | FSC-H | SSC-H | +  – | FSC-H | SSC-H | +  – | FSC-H 0 |  |
|  |  |  |  |  |  |  |  |  |  |  |  |  |  |  |  |  |
| 30 msi031904150.C06 name; msi031904150.C06 | 46000 |  | FSC-H 390 | SSC-H 110 |  | FSC-H | SSC-H |  | FSC-H | SSC-H |  | FSC-H | SSC-H |  | FSC-H 0 |  |
|  |  |  |  |  |  |  |  |  |  |  |  |  |  |  |  |  |
| 31 msi031904150.C07 name; msi031904150.C07 | 44000 |  | FSC-H 390 | SSC-H 110 |  | FSC-H | SSC-H |  | FSC-H | SSC-H |  | FSC-H | SSC-H |  | FSC-H 0 |  |
|  |  |  |  |  |  |  |  |  |  |  |  |  |  |  |  |  |
| 32 msi031904150.C08 name; msi031904150.C08 | 45000 |  | FSC-H 390 | SSC-H 100 |  | FSC-H | SSC-H |  | FSC-H | SSC-H |  | FSC-H | SSC-H |  | FSC-H 0 |  |
|  |  |  |  |  |  |  |  |  |  |  |  |  |  |  |  |  |
| 33 msi031904150.C09 name; msi031904150.C09 | 44000 |  | FSC-H 390 | SSC-H 110 |  | FSC-H | SSC-H |  | FSC-H | SSC-H |  | FSC-H | SSC-H |  | FSC-H 0 |  |
|  |  |  |  |  |  |  |  |  |  |  |  |  |  |  |  |  |
| 34 msi031904150.C10 name; msi031904150.C10 | 44000 |  | FSC-H 390 | SSC-H 110 |  | FSC-H | SSC-H |  | FSC-H | SSC-H |  | FSC-H | SSC-H |  | FSC-H 0 |  |
|  |  |  |  |  |  |  |  |  |  |  |  |  |  |  |  |  |
| 35 msi031904150.C11 name; msi031904150.C11 | 43000 |  | FSC-H 380 | SSC-H 100 |  | FSC-H | SSC-H |  | FSC-H | SSC-H |  | FSC-H | SSC-H |  | FSC-H 0 |  |
|  |  |  |  |  |  |  |  |  |  |  |  |  |  |  |  |  |
| 36 msi031904150.C12 name; msi031904150.C12 | 47000 |  | FSC-H 400 | SSC-H 110 |  | FSC-H | SSC-H |  | FSC-H | SSC-H |  | FSC-H | SSC-H |  | FSC-H 0 |  |
|  |  |  |  |  |  |  |  |  |  |  |  |  |  |  |  |  |
| 37 msi031904150.D01 name; msi031904150.D01 | 44000 |  | FSC-H 380 | SSC-H 100 |  | FSC-H | SSC-H |  | FSC-H | SSC-H |  | FSC-H | SSC-H |  | FSC-H 0 |  |
|  |  |  |  |  |  |  |  |  |  |  |  |  |  |  |  |  |
| 38 msi031904150.D02 name; msi031904150.D02 | 45000 |  | FSC-H 380 | SSC-H 100 |  | FSC-H | SSC-H |  | FSC-H | SSC-H |  | FSC-H | SSC-H |  | FSC-H 0 |  |
|  |  |  |  |  |  |  |  |  |  |  |  |  |  |  |  |  |
| 39 msi031904150.D03 name; msi031904150.D03 | 44000 |  | FSC-H 390 | SSC-H 100 |  | FSC-H | SSC-H |  | FSC-H | SSC-H |  | FSC-H | SSC-H |  | FSC-H 0 |  |
|  |  |  |  |  |  |  |  |  |  |  |  |  |  |  |  |  |
| 40 msi031904150.D04 name; msi031904150.D04 | 44000 |  | FSC-H 390 | SSC-H 100 |  | FSC-H | SSC-H |  | FSC-H | SSC-H |  | FSC-H | SSC-H |  | FSC-H 0 |  |
|  |  |  |  |  |  |  |  |  |  |  |  |  |  |  |  |  |
| 41 msi031904150.D05 name; msi031904150.D05 | 45000 |  | FSC-H 390 | SSC-H 110 |  | FSC-H | SSC-H |  | FSC-H | SSC-H |  | FSC-H | SSC-H |  | FSC-H 0 |  |
|  |  |  |  |  |  |  |  |  |  |  |  |  |  |  |  |  |
| 42 msi031904150.D06 name; msi031904150.D06 | 45000 |  | FSC-H 380 | SSC-H 110 |  | FSC-H | SSC-H |  | FSC-H | SSC-H |  | FSC-H | SSC-H |  | FSC-H 0 |  |
|  |  |  |  |  |  |  |  |  |  |  |  |  |  |  |  |  |
| 43 msi031904150.D07 name; msi031904150.D07 | 44000 | +  – | FSC-H 400 | SSC-H 110 | +  – | FSC-H | SSC-H | +  – | FSC-H | SSC-H | +  – | FSC-H | SSC-H | +  – | FSC-H 0 |  |
|  |  |  |  |  |  |  |  |  |  |  |  |  |  |  |  |  |
| 44 msi031904150.D08 name; msi031904150.D08 | 43000 |  | FSC-H 380 | SSC-H 100 |  | FSC-H | SSC-H |  | FSC-H | SSC-H |  | FSC-H | SSC-H |  | FSC-H 0 |  |
|  |  |  |  |  |  |  |  |  |  |  |  |  |  |  |  |  |
| 45 msi031904150.D09 name; msi031904150.D09 | 44000 |  | FSC-H 380 | SSC-H 110 |  | FSC-H | SSC-H |  | FSC-H | SSC-H |  | FSC-H | SSC-H |  | FSC-H 0 |  |
|  |  |  |  |  |  |  |  |  |  |  |  |  |  |  |  |  |
| 46 msi031904150.D10 name; msi031904150.D10 | 44000 |  | FSC-H 360 | SSC-H 100 |  | FSC-H | SSC-H |  | FSC-H | SSC-H |  | FSC-H | SSC-H |  | FSC-H 0 |  |
|  |  |  |  |  |  |  |  |  |  |  |  |  |  |  |  |  |
| 47 msi031904150.D11 name; msi031904150.D11 | 49000 |  | FSC-H 350 | SSC-H 100 |  | FSC-H | SSC-H |  | FSC-H | SSC-H |  | FSC-H | SSC-H |  | FSC-H 0 |  |
|  |  |  |  |  |  |  |  |  |  |  |  |  |  |  |  |  |
| 48 msi031904150.D12 name; msi031904150.D12 | 43000 |  | FSC-H 360 | SSC-H 100 |  | FSC-H | SSC-H |  | FSC-H | SSC-H |  | FSC-H | SSC-H |  | FSC-H 0 |  |
|  |  |  |  |  |  |  |  |  |  |  |  |  |  |  |  |  |
| 49 msi031904150.E01 name; msi031904150.E01 | 62000 |  | FSC-H 310 | SSC-H 90 |  | FSC-H | SSC-H |  | FSC-H | SSC-H |  | FSC-H | SSC-H |  | FSC-H 0 |  |
|  |  |  |  |  |  |  |  |  |  |  |  |  |  |  |  |  |
| 50 msi031904150.E02 name; msi031904150.E02 | 73000 |  | FSC-H 300 | SSC-H 87 |  | FSC-H | SSC-H |  | FSC-H | SSC-H |  | FSC-H | SSC-H |  | FSC-H 0 |  |
|  |  |  |  |  |  |  |  |  |  |  |  |  |  |  |  |  |
| 51 msi031904150.E03 name; msi031904150.E03 | 74000 |  | FSC-H 290 | SSC-H 82 |  | FSC-H | SSC-H |  | FSC-H | SSC-H |  | FSC-H | SSC-H |  | FSC-H 0 |  |
|  |  |  |  |  |  |  |  |  |  |  |  |  |  |  |  |  |
| 52 msi031904150.E04 name; msi031904150.E04 | 44000 |  | FSC-H 380 | SSC-H 100 |  | FSC-H | SSC-H |  | FSC-H | SSC-H |  | FSC-H | SSC-H |  | FSC-H 0 |  |
|  |  |  |  |  |  |  |  |  |  |  |  |  |  |  |  |  |
| 53 msi031904150.E05 name; msi031904150.E05 | 46000 |  | FSC-H 390 | SSC-H 100 |  | FSC-H | SSC-H |  | FSC-H | SSC-H |  | FSC-H | SSC-H |  | FSC-H 0 |  |
|  |  |  |  |  |  |  |  |  |  |  |  |  |  |  |  |  |
| 54 msi031904150.E06 name; msi031904150.E06 | 46000 |  | FSC-H 380 | SSC-H 100 |  | FSC-H | SSC-H |  | FSC-H | SSC-H |  | FSC-H | SSC-H |  | FSC-H 0 |  |
|  |  |  |  |  |  |  |  |  |  |  |  |  |  |  |  |  |
| 55 msi031904150.E07 name; msi031904150.E07 | 46000 |  | FSC-H 380 | SSC-H 100 |  | FSC-H | SSC-H |  | FSC-H | SSC-H |  | FSC-H | SSC-H |  | FSC-H 0 |  |
|  |  |  |  |  |  |  |  |  |  |  |  |  |  |  |  |  |
| 56 msi031904150.E08 name; msi031904150.E08 | 46000 |  | FSC-H 380 | SSC-H 100 |  | FSC-H | SSC-H |  | FSC-H | SSC-H |  | FSC-H | SSC-H |  | FSC-H 0 |  |
|  |  |  |  |  |  |  |  |  |  |  |  |  |  |  |  |  |
| 57 msi031904150.E09 name; msi031904150.E09 | 45000 | +  – | FSC-H 380 | SSC-H 100 | +  – | FSC-H | SSC-H | +  – | FSC-H | SSC-H | +  – | FSC-H | SSC-H | +  – | FSC-H 0 |  |
|  |  |  |  |  |  |  |  |  |  |  |  |  |  |  |  |  |
| 58 msi031904150.E10 name; msi031904150.E10 | 47000 |  | FSC-H 380 | SSC-H 100 |  | FSC-H | SSC-H |  | FSC-H | SSC-H |  | FSC-H | SSC-H |  | FSC-H 0 |  |
|  |  |  |  |  |  |  |  |  |  |  |  |  |  |  |  |  |
| 59 msi031904150.E11 name; msi031904150.E11 | 48000 |  | FSC-H 380 | SSC-H 100 |  | FSC-H | SSC-H |  | FSC-H | SSC-H |  | FSC-H | SSC-H |  | FSC-H 0 |  |
|  |  |  |  |  |  |  |  |  |  |  |  |  |  |  |  |  |
| 60 msi031904150.E12 name; msi031904150.E12 | 44000 |  | FSC-H 390 | SSC-H 100 |  | FSC-H | SSC-H |  | FSC-H | SSC-H |  | FSC-H | SSC-H |  | FSC-H 0 |  |
|  |  |  |  |  |  |  |  |  |  |  |  |  |  |  |  |  |
| 61 msi031904150.F01 name; msi031904150.F01 | 45000 |  | FSC-H 390 | SSC-H 110 |  | FSC-H | SSC-H |  | FSC-H | SSC-H |  | FSC-H | SSC-H |  | FSC-H 0 |  |
|  |  |  |  |  |  |  |  |  |  |  |  |  |  |  |  |  |
| 62 msi031904150.F02 name; msi031904150.F02 | 49000 |  | FSC-H 380 | SSC-H 100 |  | FSC-H | SSC-H |  | FSC-H | SSC-H |  | FSC-H | SSC-H |  | FSC-H 0 |  |
|  |  |  |  |  |  |  |  |  |  |  |  |  |  |  |  |  |
| 63 msi031904150.F03 name; msi031904150.F03 | 44000 |  | FSC-H 380 | SSC-H 100 |  | FSC-H | SSC-H |  | FSC-H | SSC-H |  | FSC-H | SSC-H |  | FSC-H 0 |  |
|  |  |  |  |  |  |  |  |  |  |  |  |  |  |  |  |  |
| 64 msi031904150.F04 name; msi031904150.F04 | 46000 |  | FSC-H 380 | SSC-H 100 |  | FSC-H | SSC-H |  | FSC-H | SSC-H |  | FSC-H | SSC-H |  | FSC-H 0 |  |
|  |  |  |  |  |  |  |  |  |  |  |  |  |  |  |  |  |
| 65 msi031904150.F05 name; msi031904150.F05 | 45000 |  | FSC-H 390 | SSC-H 110 |  | FSC-H | SSC-H |  | FSC-H | SSC-H |  | FSC-H | SSC-H |  | FSC-H 0 |  |
|  |  |  |  |  |  |  |  |  |  |  |  |  |  |  |  |  |
| 66 msi031904150.F06 name; msi031904150.F06 | 46000 |  | FSC-H 380 | SSC-H 110 |  | FSC-H | SSC-H |  | FSC-H | SSC-H |  | FSC-H | SSC-H |  | FSC-H 0 |  |
|  |  |  |  |  |  |  |  |  |  |  |  |  |  |  |  |  |
| 67 msi031904150.F07 name; msi031904150.F07 | 46000 |  | FSC-H 390 | SSC-H 110 |  | FSC-H | SSC-H |  | FSC-H | SSC-H |  | FSC-H | SSC-H |  | FSC-H 0 |  |
|  |  |  |  |  |  |  |  |  |  |  |  |  |  |  |  |  |
| 68 msi031904150.F08 name; msi031904150.F08 | 45000 |  | FSC-H 380 | SSC-H 100 |  | FSC-H | SSC-H |  | FSC-H | SSC-H |  | FSC-H | SSC-H |  | FSC-H 0 |  |
|  |  |  |  |  |  |  |  |  |  |  |  |  |  |  |  |  |
| 69 msi031904150.F09 name; msi031904150.F09 | 45000 |  | FSC-H 380 | SSC-H 110 |  | FSC-H | SSC-H |  | FSC-H | SSC-H |  | FSC-H | SSC-H |  | FSC-H 0 |  |
|  |  |  |  |  |  |  |  |  |  |  |  |  |  |  |  |  |
| 70 msi031904150.F10 name; msi031904150.F10 | 43000 |  | FSC-H 380 | SSC-H 100 |  | FSC-H | SSC-H |  | FSC-H | SSC-H |  | FSC-H | SSC-H |  | FSC-H 0 |  |
|  |  |  |  |  |  |  |  |  |  |  |  |  |  |  |  |  |
| 71 msi031904150.F11 name; msi031904150.F11 | 45000 | +  – | FSC-H 390 | SSC-H 100 | +  – | FSC-H | SSC-H | +  – | FSC-H | SSC-H | +  – | FSC-H | SSC-H | +  – | FSC-H 0 |  |
|  |  |  |  |  |  |  |  |  |  |  |  |  |  |  |  |  |
| 72 msi031904150.F12 name; msi031904150.F12 | 44000 |  | FSC-H 400 | SSC-H 100 |  | FSC-H | SSC-H |  | FSC-H | SSC-H |  | FSC-H | SSC-H |  | FSC-H 0 |  |
|  |  |  |  |  |  |  |  |  |  |  |  |  |  |  |  |  |
| 73 msi031904150.G01 name; msi031904150.G01 | 43000 |  | FSC-H 390 | SSC-H 110 |  | FSC-H | SSC-H |  | FSC-H | SSC-H |  | FSC-H | SSC-H |  | FSC-H 0 |  |
|  |  |  |  |  |  |  |  |  |  |  |  |  |  |  |  |  |
| 74 msi031904150.G02 name; msi031904150.G02 | 44000 |  | FSC-H 390 | SSC-H 110 |  | FSC-H | SSC-H |  | FSC-H | SSC-H |  | FSC-H | SSC-H |  | FSC-H 0 |  |
|  |  |  |  |  |  |  |  |  |  |  |  |  |  |  |  |  |
| 75 msi031904150.G03 name; msi031904150.G03 | 43000 |  | FSC-H 390 | SSC-H 100 |  | FSC-H | SSC-H |  | FSC-H | SSC-H |  | FSC-H | SSC-H |  | FSC-H 0 |  |
|  |  |  |  |  |  |  |  |  |  |  |  |  |  |  |  |  |
| 76 msi031904150.G04 name; msi031904150.G04 | 45000 |  | FSC-H 390 | SSC-H 110 |  | FSC-H | SSC-H |  | FSC-H | SSC-H |  | FSC-H | SSC-H |  | FSC-H 0 |  |
|  |  |  |  |  |  |  |  |  |  |  |  |  |  |  |  |  |
| 77 msi031904150.G05 name; msi031904150.G05 | 46000 |  | FSC-H 380 | SSC-H 100 |  | FSC-H | SSC-H |  | FSC-H | SSC-H |  | FSC-H | SSC-H |  | FSC-H 0 |  |
|  |  |  |  |  |  |  |  |  |  |  |  |  |  |  |  |  |
| 78 msi031904150.G06 name; msi031904150.G06 | 47000 |  | FSC-H 380 | SSC-H 100 |  | FSC-H | SSC-H |  | FSC-H | SSC-H |  | FSC-H | SSC-H |  | FSC-H 0 |  |
|  |  |  |  |  |  |  |  |  |  |  |  |  |  |  |  |  |
| 79 msi031904150.G07 name; msi031904150.G07 | 47000 |  | FSC-H 390 | SSC-H 100 |  | FSC-H | SSC-H |  | FSC-H | SSC-H |  | FSC-H | SSC-H |  | FSC-H 0 |  |
|  |  |  |  |  |  |  |  |  |  |  |  |  |  |  |  |  |
| 80 msi031904150.G08 name; msi031904150.G08 | 47000 |  | FSC-H 380 | SSC-H 100 |  | FSC-H | SSC-H |  | FSC-H | SSC-H |  | FSC-H | SSC-H |  | FSC-H 0 |  |
|  |  |  |  |  |  |  |  |  |  |  |  |  |  |  |  |  |
| 81 msi031904150.G09 name; msi031904150.G09 | 47000 |  | FSC-H 380 | SSC-H 100 |  | FSC-H | SSC-H |  | FSC-H | SSC-H |  | FSC-H | SSC-H |  | FSC-H 0 |  |
|  |  |  |  |  |  |  |  |  |  |  |  |  |  |  |  |  |
| 82 msi031904150.G10 name; msi031904150.G10 | 46000 |  | FSC-H 380 | SSC-H 100 |  | FSC-H | SSC-H |  | FSC-H | SSC-H |  | FSC-H | SSC-H |  | FSC-H 0 |  |
|  |  |  |  |  |  |  |  |  |  |  |  |  |  |  |  |  |
| 83 msi031904150.G11 name; msi031904150.G11 | 47000 |  | FSC-H 380 | SSC-H 100 |  | FSC-H | SSC-H |  | FSC-H | SSC-H |  | FSC-H | SSC-H |  | FSC-H 0 |  |
|  |  |  |  |  |  |  |  |  |  |  |  |  |  |  |  |  |
| 84 msi031904150.G12 name; msi031904150.G12 | 46000 |  | FSC-H 400 | SSC-H 110 |  | FSC-H | SSC-H |  | FSC-H | SSC-H |  | FSC-H | SSC-H |  | FSC-H 0 |  |
|  |  |  |  |  |  |  |  |  |  |  |  |  |  |  |  |  |
| 85 msi031904150.H01 name; msi031904150.H01 | 47000 | +  – | FSC-H 380 | SSC-H 100 | +  – | FSC-H | SSC-H | +  – | FSC-H | SSC-H | +  – | FSC-H | SSC-H | +  – | FSC-H 0 |  |
|  |  |  |  |  |  |  |  |  |  |  |  |  |  |  |  |  |
| 86 msi031904150.H02 name; msi031904150.H02 | 50000 |  | FSC-H 370 | SSC-H 100 |  | FSC-H | SSC-H |  | FSC-H | SSC-H |  | FSC-H | SSC-H |  | FSC-H 0 |  |
|  |  |  |  |  |  |  |  |  |  |  |  |  |  |  |  |  |
| 87 msi031904150.H03 name; msi031904150.H03 | 49000 |  | FSC-H 380 | SSC-H 100 |  | FSC-H | SSC-H |  | FSC-H | SSC-H |  | FSC-H | SSC-H |  | FSC-H 0 |  |
|  |  |  |  |  |  |  |  |  |  |  |  |  |  |  |  |  |
| 88 msi031904150.H04 name; msi031904150.H04 | 51000 |  | FSC-H 380 | SSC-H 100 |  | FSC-H | SSC-H |  | FSC-H | SSC-H |  | FSC-H | SSC-H |  | FSC-H 0 |  |
|  |  |  |  |  |  |  |  |  |  |  |  |  |  |  |  |  |
| 89 msi031904150.H05 name; msi031904150.H05 | 47000 |  | FSC-H 380 | SSC-H 100 |  | FSC-H | SSC-H |  | FSC-H | SSC-H |  | FSC-H | SSC-H |  | FSC-H 0 |  |
|  |  |  |  |  |  |  |  |  |  |  |  |  |  |  |  |  |
| 90 msi031904150.H06 name; msi031904150.H06 | 49000 |  | FSC-H 380 | SSC-H 100 |  | FSC-H | SSC-H |  | FSC-H | SSC-H |  | FSC-H | SSC-H |  | FSC-H 0 |  |
|  |  |  |  |  |  |  |  |  |  |  |  |  |  |  |  |  |
| 91 msi031904150.H07 name; msi031904150.H07 | 47000 |  | FSC-H 380 | SSC-H 100 |  | FSC-H | SSC-H |  | FSC-H | SSC-H |  | FSC-H | SSC-H |  | FSC-H 0 |  |
|  |  |  |  |  |  |  |  |  |  |  |  |  |  |  |  |  |
| 92 msi031904150.H08 name; msi031904150.H08 | 51000 |  | FSC-H 380 | SSC-H 100 |  | FSC-H | SSC-H |  | FSC-H | SSC-H |  | FSC-H | SSC-H |  | FSC-H 0 |  |
|  |  |  |  |  |  |  |  |  |  |  |  |  |  |  |  |  |
| 93 msi031904150.H09 name; msi031904150.H09 | 50000 |  | FSC-H 370 | SSC-H 100 |  | FSC-H | SSC-H |  | FSC-H | SSC-H |  | FSC-H | SSC-H |  | FSC-H 0 |  |
|  |  |  |  |  |  |  |  |  |  |  |  |  |  |  |  |  |
| 94 msi031904150.H10 name; msi031904150.H10 | 50000 |  | FSC-H 380 | SSC-H 100 |  | FSC-H | SSC-H |  | FSC-H | SSC-H |  | FSC-H | SSC-H |  | FSC-H 0 |  |
|  |  |  |  |  |  |  |  |  |  |  |  |  |  |  |  |  |
| 95 msi031904150.H11 name; msi031904150.H11 | 54000 |  | FSC-H 370 | SSC-H 100 |  | FSC-H | SSC-H |  | FSC-H | SSC-H |  | FSC-H | SSC-H |  | FSC-H 0 |  |
|  |  |  |  |  |  |  |  |  |  |  |  |  |  |  |  |  |
| 96 msi031904150.H12 name; msi031904150.H12 | 31000 |  | FSC-H 200 | SSC-H 58 |  | FSC-H | SSC-H |  | FSC-H | SSC-H |  | FSC-H | SSC-H |  | FSC-H 0 |  |
|  |  |  |  |  |  |  |  |  |  |  |  |  |  |  |  |  |

Frames 1-14
Frames 15-28
Frames 29-42
Frames 43-56
Frames 57-70
Frames 71-84
Frames 85-96
